# Predictive value of neurophysiological monitoring during posterior communicating artery aneurysm clipping for postoperative neurological deficits

**DOI:** 10.3389/fsurg.2022.1043428

**Published:** 2023-01-06

**Authors:** Fengjiao Tang, Shifang Li, Juntao Wang, Wanzhong Tang, Yugong Feng

**Affiliations:** ^1^ Department of Neurosurgery, Affiliated Hospital of Qingdao University, Qingdao, China; ^2^ Department of Anesthesiology, Affiliated Hospital of Qingdao University, Qingdao, China

**Keywords:** intraoperative neurophysiological monitoring, motor evoked potential, somatosensory evoked potential, posterior communicating artery, aneurysm clipping, temporary clipping

## Abstract

**Objective:**

This study aimed to evaluate the diagnostic effect of intraoperative neurophysiological monitoring in identifying intraoperative ischemic events and predicting postoperative neurological dysfunction during PCoA aneurysm clipping, as well as to explore the safe duration of intraoperative temporary clipping of the parent artery.

**Methods:**

All 71 patients with PCoA aneurysm underwent craniotomy and aneurysm clipping. MEP and SSEP were used for monitoring during operation to evaluate the influence of MEP/SSEP changes on postoperative neurological function. Receiver operating characteristic (ROC) curve analysis was used to calculate optimal duration of intraoperative temporary clipping.

**Results:**

Patients with intraoperative MEP/SSEP changes were more likely to develop short-term and long-term neurological deficits than those without MEP/SSEP changes (*P* < 0.05). From the ROC curve analysis, the safe time from the initiation of temporary clipping during the operation to the early warning of neurophysiological monitoring was 4.5 min (AUC = 0.735, 95%CI 0.5558-0.912). Taking 4.5 min as the dividing line, the incidence of short-term and long-term neurological dysfunction in patients with temporary clipping >4.5 min was significantly higher than that in patients with temporary clipping ≤4.5 min (*P* = 0.015, *P* = 0.018).

**Conclusion:**

Intraoperative MEP/SSEP changes are significantly associated with postoperative neurological dysfunction in patients with PCoA aneurysms. The optimal duration of temporary clipping of the parent artery during posterior communicating aneurysm clipping was 4.5 min under neurophysiological monitoring.

## Introduction

Posterior communicating artery (PCoA) aneurysms are the second most common aneurysm, accounting for 25 percent of all intracranial aneurysms and 50 percent of all internal carotid artery aneurysms (1, 2). PCoA aneurysms are more prone to rupture than intracranial aneurysms located elsewhere (1). The development of PCoA varies greatly in different individuals, and this difference makes PCoA aneurysms either the most manageable intracranial aneurysm or the most difficult to intervene. At the same time, PCoA sends out multiple important branch vessels to supply blood to important structures such as the surrounding thalamus, caudate nucleus, internal capsule, etc. During craniotomy aneurysm clipping, improper operation affects the normal blood supply of PCoA and its branches, resulting in Complications such as postoperative ischemic brain injury in patients (3). Since the 1970s, intraoperative neurophysiological monitoring (IONM) technology has been used in aneurysm surgery to avoid neurological deficits caused by transient postoperative ischemia (4). At present, IONM is widely used in intracranial aneurysm clipping, among which motor evoked potential (MEP) and somatosensory evoked potential (SSEP) are safe and effective methods for real-time monitoring of acute cerebral ischemic injury (5). In addition, MEP and SSEP monitoring plays an important role in preventing ischemic brain injury caused by occlusion of parent artery for too long during surgery. In recent decades, many teams have studied the optimal safe time for temporary clipping of the parent artery during surgery, but so far, there is still no unified definition standard (6–8). In clinical practice, we have found that many studies are imprecise in defining the safe duration of temporary clipping, because aneurysms in different locations tolerate cerebral ischemia differently. Therefore, the ideal standard for predicting intraoperative cerebral ischemia should be determined according to the location of the aneurysm and the actual situation during the operation. In this study, we chose PCoA aneurysm as the entry point to study the application of IONM to analyze the specific correlation between the safe duration of temporary clipping and the location of the aneurysm, in order to provide more information for the clinical application of IONM. For reference, the specific content is reported as follows.

## Materials and methods

### Patient population

A total of 71 patients with PCoA aneurysms in neurosurgery of Qingdao University Affiliated Hospital from April 2019 to May 2022 were retrospectively included, and were diagnosed by preoperative CT, CTA and/or DSA. All patients underwent craniotomy to clip the aneurysm. There were 14 males and 57 females, ranging in age from 37 to 81 years old, with an average of (61.62 ± 9.35) years old. Aneurysm rupture occurred in 29 cases, and 42 cases did not rupture. Aneurysm size: <5 mm in 29 cases, 5–10 mm in 31 cases, and 10–25 mm in 11 cases. There were 31 patients with temporary clipping of parent artery during operation, and 40 patients without temporary clipping. This study was approved by the ethics committee of our hospital. All patients gave informed consent to participate in the study.

### Surgical procedure

The microsurgical approach is the pterional approach. After the aneurysm is exposed through fine dissection, the neck of the aneurysm is carefully separated and identified, and the parent artery is temporarily clipped according to the specific situation during the operation. Neurophysiological monitoring technology is applied throughout the operation. A decrease in MEP and SSEP amplitude of more than 50% is considered a significant change requiring early warning and intervention after excluding anesthetic and physiological effects. When the amplitude of MEP and SSEP monitoring decreases to the alarm threshold, the surgeon will be informed immediately, and the cause of ischemia will be determined according to the actual operation during the operation, and the operation will be stopped and adjusted accordingly, including releasing temporary clips, releasing brain retractors, adjusting aneurysm clips, applying papaverine wet compresses and other surgical operations, and continuing the operation after the abnormal MEP and (or) SSEP return to the baseline level.

### Intraoperative MEP and SSEP monitoring

All patients were treated with intravenous inhalation anesthesia. Typically, intraoperative muscle relaxants are not administered after induction of anesthesia. The Cadwell Cascade 32-lead intraoperative evoked potential monitoring system was used, and the corresponding IONM scheme was designed according to the location of the aneurysm. According to the international EEG 10/20 standard, electrodes are placed and parameters are set: (1) SSEP monitoring, the recording electrodes are spiral electrodes, the upper limbs are placed at C3′ and C4′, the lower limbs are placed at Cz, and the reference electrodes are placed at FPz. The stimulation electrodes were needle electrodes, the upper limb was the median nerve of the wrist, and the lower limb was the posterior tibial nerve of the ankle. Constant current monophasic pulse stimulation was used, the stimulation frequency was 2.79 Hz, the stimulation interval was 200 *μ*s, the current stimulation intensity was 15–35 mA, the sensitivity was 1–10 μV, the band-pass was 30–500 Hz, the duration was 50–100 ms, and the average superposition was 100–200 times. (2) MEP monitoring, the stimulation electrodes are placed on C3, C4 or C1, C2, the two act as reference electrodes for each other, the recording electrodes are placed on the upper limb abductor pollicis brevis and the lower limb adductor muscle, and short series of electrical stimulation is used, generally given 5–8 single series of stimulation, each single stimulation duration 50 ms, stimulation interval 1–2 ms, stimulation voltage 100–400 V, sensitivity 50–200 *μ*V, bandpass 100–3000 Hz, analysis time 100 ms. MEP and SSEP were recorded once after anesthesia and before opening of the dura to obtain baseline values. After the dura mater was opened, MEP and SSEP were recorded routinely every 5 min, and MEP and SSEP should be recorded frequently when the operation proceeded to critical steps, such as temporary clipping of the parent artery and adjustment of aneurysm clips. In the event of a warning, the surgeon should take appropriate measures, such as lifting the temporary occlusion or adjusting the clip placement, to restore the MEP and SSEP amplitudes and prevent ischemic injury.

### Outcome criteria and evaluation

The curative effect evaluation of 71 patients was based on 1 day and 3 months after surgery, and the score (Glasgow Outcome Scare, GOS) was used to evaluate the prognosis. The higher the score, the better the prognosis and vice versa. Neurological deficits at 1 day after surgery were defined as short-term neurological deficits, while neurological deficits at 3 months postoperatively were defined as long-term neurological deficits. Postoperative evaluation of all patients after surgery was performed by a neurosurgeon and a neurophysiological monitoring physician.

### Statistical analysis

Statistical analysis was performed using SPSS 26.0 software. Measurement data were expressed as mean ± standard deviation $\left(\bar{x}\pm s\right)$, and comparisons between groups were expressed by t test or nonparametric test; enumeration data were expressed by number of cases or percentages, and comparisons between groups were expressed by *χ*^2^ test or Fisher's exact test. *P* < 0.05 was considered to be statistically significant. Receiver operating characteristic (ROC) curve analysis was used to determine the optimal duration of intraoperative temporary clipping and ischemia tolerance time for changes in MEP/SSEP.

## Results

### The relationship between intraoperative MEP/SSEP changes and clinical characteristics of patients with PCoA aneurysm

Of all 71 patients with PCoA aneurysm, 21 (29.6%) showed IONM changes, 17(23.9%) showed MEP changes, 11(15.5%) showed SSEP changes, and 7 (9.9%) showed combined MEP and SSEP changes. The characteristics of patients classified according to the occurrence of MEP/SSEP changes are summarized in Table 1. There were no differences in gender, age, aneurysm size, and rupture of patients with or without MEP/SSEP changes, but the incidence of temporary clipping in patients with MEP/SSEP changes was significantly higher than that in patients without changes (*P* < 0.05).

**Table 1:** Clinical characteristics of 71 patients with posterior communicating artery aneurysm.

Parameter	Total (*n* = 71)	MEP			SSEP		
		MEP changes (*n* = 17)	No MEP changes (*n* = 54)	*P*	SSEP changes (*n* = 11)	No SSEP changes (*n* = 60)	*P*
Age				0.258			0.129
Mean ± SD	61.62 ± 9.35	63.06 ± 10.31	61.17 ± 9.09		61.73 ± 6.44	61.60 ± 9.84	
Gender				0.806			0.889
Male	14	3	11		2	12	
Female	57	14	43		9	48	
SAH				0.245			0.735
Yes	29	9	20		5	24	
No	42	8	34		6	36	
Size (mm)				0.095			0.468
<5	29	5	25		4	25	
5–10	31	7	23		4	27	
10–25	11	5	6		3	8	
Temporary clipping				0.000			0.006
Yes	31	14	17		9	22	
No	40	3	37		2	38	

### Intraoperative MEP/SSEP monitoring results and postoperative neurological dysfunction

Of the 17 patients with PCoA aneurysms with significant intraoperative MEP changes, 8 (47.1%) had short-term neurological deficits and 4 (23.5%) had long-term neurological deficits. Of the 54 patients with no intraoperative MEP change, 3 (5.6%) had short-term neurological deficits and 1 (1.9%) had long-term neurological deficits. Patients with intraoperative MEP changes were more likely to develop short-term and long-term neurological deficits than those without MEP changes (*P* < 0.001, *P* < 0.05, Fisher's exact probability, Table 2). Among 11 aneurysm patients with significant intraoperative SSEP changes, 5 (45.5%) had short-term neurological deficits and 3 (27.3%) long-term neurological deficits. Of the 60 patients with no intraoperative SSEP changes, 6 (10.0%) had short-term neurological deficits and 2 (3.3%) had long-term neurological deficits. Overall, the conclusions are consistent with those obtained from the MEP monitoring described above (*P* < 0.05, chi-square test, *P* < 0.05, Fisher's exact probability, Table 3).

**Table 2:** Relationship between intraoperative MEP and postoperative neurological dysfunction and GOS score.

	MEP changes (*n*, %)	No MEP changes (*n*, %)	Total (*n* = 71)	*P*
Short-term neurological dysfunction (*n*,%)				<0.001
Yes	8 (47.1%)	3 (5.6%)	11	
No	9 (52.9%)	51 (94.4%)	60	
Long-term neurological dysfunction (*n*,%)				<0.05
Yes	4 (23.5%)	1 (1.9%)	5	
No	13 (76.5%)	53 (98.1%)	66	
GOS				
1-day follow-up	4.24 ± 0.97	4.89 ± 0.46		<0.001
3-month follow-up	4.65 ± 0.70	4.98 ± 0.14		<0.05

**Table 3:** Relationship between intraoperative SSEP and postoperative neurological dysfunction and GOS score.

	SSEP changes (*n*, %)	No SSEP changes (*n*, %)	Total (*n*, %)	*P*
Short-term neurological dysfunction (*n*,%)				<0.05
Yes	5 (45.5%)	6 (10.0%)	11	
No	6 (54.5%)	54 (90.0%)	60	
Long-term neurological dysfunction (*n*,%)				<0.05
Yes	3 (27.3%)	2 (3.3%)	5	
No	8 (72.7%)	58 (96.7%)	66	
GOS				
1-day follow-up	4.00 ± 1.00	4.87 ± 0.50		<0.001
3-month follow-up	4.64 ± 0.67	4.95 ± 0.29		<0.05

**Figure 1:**
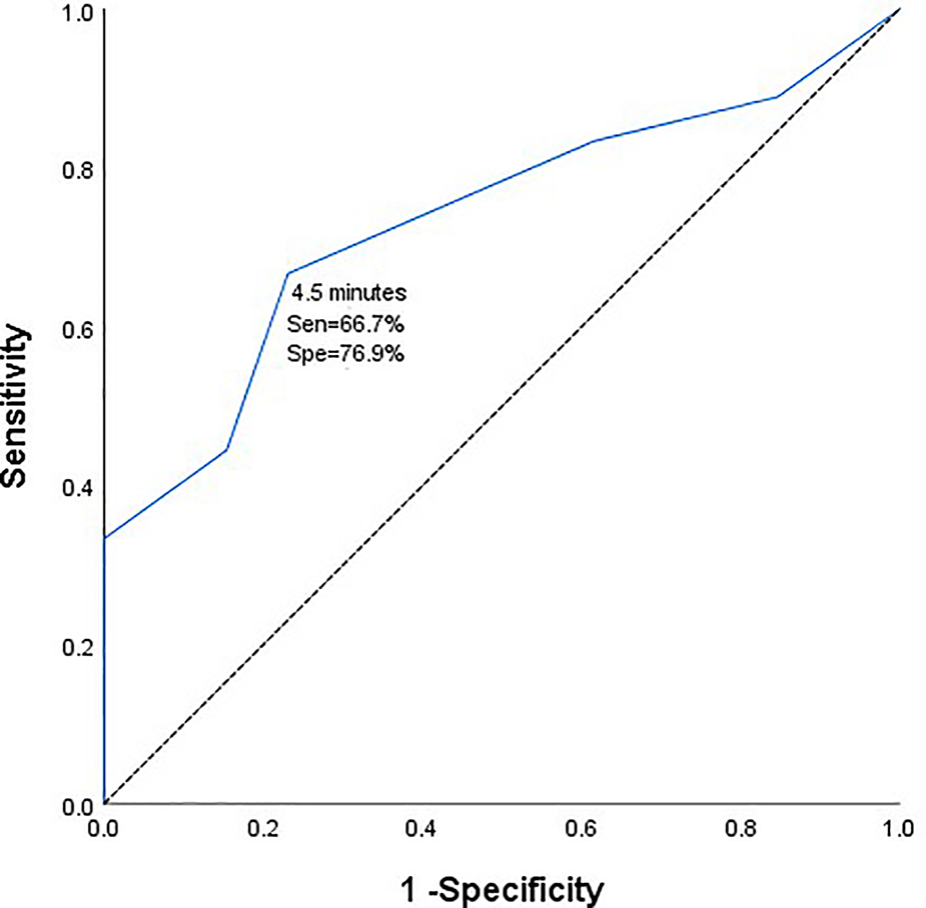
Receiver operating characteristic curve of duration of intraoperative temporary clipping and electrophysiological monitoring and warning.

There were 31 patients with temporary clipping during operation. From the ROC curve analysis, it can be seen that the safe duration from the initiation of temporary clipping to the occurrence of MEP/SSEP warning was 4.5 min respectively (area under the curve (AUC) = 0.735, 95% confidence interval [CI] 0.558–0.912, Figure 1). The duration of temporary clipping was longer than 4.5 min in 15 patients, of which 7 (46.7%) patients developed short-term neurological deficits after surgery, and 5 (33.3%) patients developed long-term functional deficits. Temporary clipping time of 16 patients was within 4.5 min, of which only 1 patient (6.25%) developed short-term neurological dysfunction, and none (0%) developed long-term neurological dysfunction. The odds of postoperative short-term and long-term neurological dysfunction were significantly higher in patients with temporary clipping >4.5 min than in patients with temporary clipping ≤4.5 min (*P* = 0.015, *P* = 0.018, Fisher's exact probability, Table 4).

**Table 4:** Relationship between temporary clipping time and postoperative neurological dysfunction.

	Temporary clipping duration		Total (*n* = 31)	*P*
	≤4.5 min	>4.5 min		
Short-term neurological dysfunction (*n*,%)				0.015
Yes	1 (6.25%)	7 (46.7%)	8	
No	15 (93.75%)	8 (53.3%)	23	
Long-term neurological dysfunction (*n*,%)				0.018
Yes	0 (0%)	5 (33.3%)	5	
No	16 (100%)	10 (66.7%)	26	

### Intraoperative MEP/SSEP changes and GOS score

The GOS scores at 1 day (short-term) and 3 months (long-term) after surgery were compared in patients with or without intraoperative MEP/SSEP changes. On 1 day after surgery, patients with no intraoperative MEP change had significantly higher GOS scores than those with MEP change (4.89 ± 0.46 vs. 4.24 ± 0.97, *P* < 0.001, Mann–Whitney *U* test, Table 2). At 3-momth follow-up, the results were the same (4.98 ± 0.14 vs. 4.65 ± 0.70, *P* < 0.05, Mann–Whitney *U* test, Table 2). For intraoperative SSEP monitoring follow-up results, 1 day after surgery, SSEP did not change vs. SSEP changed (4.87 ± 0.50 vs. 4.00 ± 1.00, *P* < 0.001, Mann–Whitney *U* test, Table 3). At 3-month follow-up, there was no change in SSEP vs. change in SSEP (4.95 ± 0.29 vs. 4.64 ± 0.67, *P* < 0.05, Mann–Whitney *U* test, Table 3).

## Discussion

At present, MEP and SSEP monitoring are commonly used electrophysiological monitoring techniques in craniotomy aneurysm clipping, and are also important means to predict whether patients will develop new neurological dysfunction after surgery. Intraoperative cerebral ischemia is an important cause of postoperative neurological dysfunction and even death. Intraoperative continuous SSEP and MEP monitoring can evaluate the integrity of the nervous system in real time, detect intraoperative cerebral ischemia and brain tissue damage in time, and then take corresponding intervention measures, which greatly reduces postoperative complications. In our study, the incidence of postoperative new neurological dysfunction was 47.1% (8/17) and 5.6% (3/54) in patients with and without intraoperative MEP changes, respectively, and the GOS score was 4.24 ± 0.97 and 4.89 ± 0.46. For SSEP, the incidence was 45.5% (5/11) and 10.0% (6/60), and the GOS scores were 4.00 ± 1.00 and 4.87 ± 0.50, respectively. Our analysis showed that intraoperative MEP and SSEP changes were significantly associated with new postoperative neurological deficits and lower quality of life. Our review of the literature found that other research groups have come to the same conclusion. Nakagawa I et al. believed that intraoperative MEP changes could reliably predict postoperative neurological dysfunction, in which the permanent loss of intraoperative MEP would lead to severe postoperative neurological dysfunction (9). Guo D et al. reported that postoperative neurological function and quality of life in patients with significant changes in MEP were significantly lower than those in patients with no changes in MEP (10). A meta-analysis by Thirumala PD et al. showed that patients with intraoperative changes in SSEP were 7 times more likely to develop postoperative neurological dysfunction than those without changes (11). From this we can see that patients with significant intraoperative changes in MEP and SSEP have a significantly increased risk of developing new neurological deficits after surgery and have a worse prognosis.

During clipping of a PCoA aneurysm, temporary clipping of the parent artery is often required to completely stop blood flow to the aneurysm. The risk of postoperative ischemia-induced neurological deficits greatly increases with prolonged temporary clipping. IONM can monitor patients for intraoperative cerebral cortical ischemia and subcortical ischemia during the temporary clipping of the blood supply of the parent artery (12). Temporary clipping has been used in intracranial aneurysm clipping since 1928, and despite evolving medical techniques and surgical strategies, controversy over the maximum allowable duration and safety of temporary clipping remains unanswered (13). In a noncontemporaneous prospective analysis of 132 patients with aneurysms at Massachusetts General Hospital from 1991 to 1993, the safe duration of intraoperative temporary clipping should not exceed 20 min (14). In 2014, Griessenauer CJ et al. came to a similar conclusion. They concluded that the longest duration of intraoperative temporary clipping in SAH and unruptured aneurysms was 19.4 min and 16.1 min, respectively, and within these timeframes, patients did not experience neurological deficits postoperatively (15, 16). With the advancement of medical technology and the application of intraoperative neurophysiological techniques, we can detect ischemic changes faster before clinical manifestations in patients, thereby improving the safety of temporary clipping. A 2020 study by Kameda et al. reported a safe time of 5 min for the longest interim clip under intraoperative neurophysiological monitoring (17). Our team published a report in 2021 that temporary clipping of the parent artery should not exceed 6 min. In this study, we analyzed the safe duration from the initiation of temporary clipping to the change in intraoperative MEP/SSEP monitoring of 4.5 min for the specificity of PCoA aneurysm location (AUC = 0.735, 95%CI 0.558-0.912). Moreover, the incidence of short-term and long-term neurological dysfunction was significantly higher in patients with temporary clipping >4.5 min than in patients with temporary clipping ≤4.5 min (*P* = 0.015, *P* = 0.018, Fisher's exact probability). From this we can see that the safe time of temporary clipping is different for different locations and characteristics of aneurysms, and we believe that 4.5 min is the best cut-off value for temporary clipping in PCoA aneurysm clipping. This finding extends our past findings and represents a new step toward an individualized prediction system for postoperative neurological dysfunction in patients with aneurysms.

In this study, MEP/SSEP changes occurred in 21 cases, including 18 cases due to prolonged temporary clipping of the internal carotid artery during surgery, 3 cases due to mis-clamping of important perforating vessels of PCoA, and 11 cases with new neurological dysfunction after operation. It can be seen that IONM plays an important role in predicting intraoperative cerebral ischemia and preventing new postoperative neurological dysfunction. It is worth mentioning that we found that only significant changes in MEP occurred in 3 patients who had mis-clamped important perforators due to PCoA, but no changes in SSEP. This also confirms to some extent that MEP monitoring is superior to SSEP in predicting motor impairment caused by subcortical perforator ischemia (18). However, MEP is less sensitive than SSEP in predicting cerebral cortical ischemia, and MEP is greatly affected by anesthesia factors, and its waveform is less stable than SSEP. In the current monitoring of intracranial aneurysm clipping surgery, MEP as a supplement to SEP, combined monitoring of the two can reduce the probability of cerebral cortical and subcortical ischemia during operation, and provide a reliable theoretical basis for the time limit of parent artery occlusion during operation.

## Conclusions

Regarding the application of IONM in PCoA aneurysms, we have the following three experiences: First, one of the goals of this study was to determine the relationship between intraoperative MEP and SSEP changes and postoperative neurological dysfunction in patients. Patients with changes in MEP and SSEP had a significantly higher risk of postoperative neurological dysfunction than those without changes. Second, the critical thresholds for intraoperative changes from temporary clipping to intraoperative neurophysiological monitoring were 4.5 min, respectively, a finding that has important clinical implications. Third, at present, most neurosurgeons do not have a comprehensive understanding of the application value of IONM in craniotomy and artery clipping. Predictive value, while ignoring the actual application of IONM in predicting ischemic events caused by other reasons (such as excessive clipping of the aneurysm neck, associated clipping of collateral vessels, and incorrect clipping of perforating arteries, etc.) clinical significance.

## Data Availability

The original contributions presented in the study are included in the article/Supplementary Material, further inquiries can be directed to the corresponding author.
